# Serum testosterone and cardiometabolic risk in Nigerian men with type 2 diabetes: a cross−sectional study

**DOI:** 10.3389/fendo.2026.1843249

**Published:** 2026-06-16

**Authors:** Ezekiel Musa, Esteban Salazar-Petres, Glory Chinuru Musa, Fatima Bello, Adamu Girei Bakari

**Affiliations:** 1 Department of Internal Medicine, Kaduna State University, Kaduna, Nigeria; 2 North Cumbria Integrated Care NHS Foundation Trust, Carlisle, United Kingdom; 3 Escuela de Obstetricia, Facultad Ciencias Para el Cuidado de la Salud, Universidad San Sebastián, Valdivia, Chile; 4 National Ear Care Centre, Kaduna, Nigeria; 5 Department of Medicine, Ahmadu Bello University, Zaria, Kaduna State, Nigeria

**Keywords:** ASCVD risk score, cardiometabolic risk, low testosterone, Nigerian men, type 2 diabetes

## Abstract

**Introduction:**

Type 2 diabetes mellitus (T2DM) increasingly burdens cardiometabolic health in Nigerian men, with evidence indicating testosterone deficiency may worsen atherosclerotic cardiovascular disease (ASCVD) risk. Despite the high rates of hypogonadism in T2DM men, limited studies have examined how low testosterone is linked with cardiometabolic risk. This study assessed the associations between serum testosterone and cardiometabolic risk in Nigerian men with T2DM.

**Methods:**

In this cross-sectional study, 537 adult men were recruited, 358 with T2DM and 179 non-diabetic controls. Participants were stratified by T2DM and testosterone levels. Clinical, anthropometric, and biochemical measurements, including lipid and serum testosterone, were obtained. The 10-year ASCVD risk was estimated using ACC/AHA equations. Data were analyzed using two-way ANOVA, Pearson correlations, and multivariable regression.

**Results:**

Men with T2DM had significantly higher ASCVD 10-year score than non-diabetic controls, independent of testosterone status (P_T2DM_<0.0001). Hypogonadal T2DM men exhibited higher BMI, central obesity, total and LDL cholesterol, and lower HDL cholesterol. In multivariable analyses within the T2DM cohort, serum testosterone was not independently associated with the ASCVD risk score or any individual cardiometabolic risk factor.

**Conclusion:**

Men with T2DM have a higher estimated 10-year ASCVD risk than non-diabetic controls, irrespective of testosterone status, with T2DM men with low testosterone showing a more adverse cardiometabolic profile. However, testosterone is not an independent predictor of ASCVD risk after multivariable adjustment, suggesting that T2DM-related metabolic risk factors are the dominant drivers of calculated cardiovascular risk. Longitudinal studies are required to establish causality and clinical relevance.

## Introduction

Type 2 diabetes mellitus (T2DM) represents a growing public health challenge in Nigeria and across sub-Saharan Africa (1, 2). The condition is associated with substantial morbidity and mortality, primarily driven by macrovascular complications, including atherosclerotic cardiovascular disease (ASCVD) (3). Nigerian men with T2DM face a particularly elevated cardiovascular risk profile, compounded by the high prevalence of traditional risk factors such as hypertension, dyslipidemia, and central obesity (4). The metabolic syndrome, a constellation of cardiovascular risk factors, has been documented in 44% to 85.8% of T2DM with low testosterone in African studies (5, 6). Understanding modifiable risk factors that contribute to ASCVD in this population is critical for developing targeted prevention and intervention strategies. Substantial global evidence demonstrates a bidirectional relationship between testosterone deficiency and T2DM in men. Multiple studies have documented significantly lower testosterone levels in men with T2DM compared to non-diabetic controls, with hypogonadism prevalence ranging from 30% to 80.4% in diabetic populations (7–9). The pathophysiological mechanisms linking low testosterone to diabetes include increased visceral adiposity, insulin resistance, chronic inflammation, and endothelial dysfunction (10). Prospective studies have shown that low testosterone concentrations predict not only the development of T2DM but also adverse metabolic outcomes, including poor glycemic control and increased cardiovascular events (11). This relationship appears independent of traditional risk factors such as age, body mass index, and lipid profiles.

Direct evidence linking testosterone deficiency to ASCVD in men with T2DM has emerged from multiple imaging and clinical outcome studies. Landmark investigations have demonstrated inverse correlations between testosterone levels and carotid intima-media thickness (IMT), a validated marker of subclinical atherosclerosis. A significant negative correlation was reported between free testosterone and carotid IMT in Japanese men with T2DM (12). Similarly, another study found a stronger inverse relationship between total testosterone and carotid IMT in men with T2DM, with low-testosterone groups also exhibiting higher carotid plaque and impaired endothelial function. Low serum testosterone concentrations have been demonstrated to predict incident acute myocardial infarction in men with T2DM (13). A systematic review and meta-analysis synthesized data from 3, 467 men with T2DM, reporting a pooled relative risk of 1.24 (95% CI 0.94- 1.63) for coronary artery disease in those with low testosterone, though this did not reach statistical significance (14). More recently, it was established that overt hypogonadism is an independent cardiovascular risk factor in T2DM men, with macrovascular complications occurring significantly more frequently in hypogonadal men (15).

Nigerian studies have consistently documented high rates of testosterone deficiency in men with T2DM, with prevalence estimates ranging from 30% to 80.4%. A study conducted at Gbagada General Hospital in Lagos reported a 35.3% prevalence of testosterone deficiency syndrome among 203 men with T2DM, including 18.3% with mild deficiency and 17% with severe deficiency (5). A parallel study in Lagos, Nigeria, found a 36% prevalence of testosterone deficiency syndrome, though notably, no significant correlation was observed between testosterone levels and metabolic syndrome parameters in this cohort (5, 16). More recent Nigerian investigations have provided mechanistic insights. Significantly lower calculated free testosterone in 104 Nigerian men with T2DM than in controls, with lower testosterone associated with reduced insulin sensitivity and poorer glycemic control, has been demonstrated (17). Studies reported a mean total testosterone of 8.79 ± 3.35 nmol/L in 358 Nigerian men with T2DM, compared with 15.41 ± 3.79 nmol/L in controls (p < 0.001), identifying triglycerides and HDL cholesterol as independent correlates of hypogonadism (9, 18). Another study found 30% hypogonadism prevalence among diabetic men in South-Western Nigeria, with 66.7% of hypogonadal diabetics being obese, highlighting the amplifying effect of obesity on testosterone reduction (19). In South Africa, the only African study directly linking low testosterone to existing cardiovascular disease was conducted, reporting approximately 50% prevalence of low total testosterone in 150 male diabetic patients aged 50 years or older, with waist circumference and known cardiovascular disease independently associated with low testosterone (20). Another cross-sectional study reported a prevalence of 35.8% of low total testosterone in South African men with T2DM, with inverse correlations to body mass index, waist circumference, and metabolic syndrome components (6). Recent Ethiopian data reported 30.3% hypogonadism prevalence in men with T2DM versus 7.2% in controls, with hyperglycemia, low HDL, obesity, and age as independent predictors (21). Another study found 43.5% testosterone deficiency prevalence in Afro-Caribbean men with T2DM, occurring more frequently in subjects with macrovascular disease (22).

Multiple interconnected pathophysiological mechanisms explain the relationship between testosterone deficiency and increased ASCVD risk in men with T2DM. Low testosterone promotes visceral adiposity and insulin resistance, creating a metabolic milieu conducive to atherosclerosis (10). Testosterone deficiency is associated with adverse lipid profiles, particularly elevated triglycerides and reduced HDL cholesterol, as demonstrated in Nigerian populations (18). Endothelial dysfunction, a critical early step in atherogenesis, is more pronounced in diabetic men with low testosterone, as evidenced by impaired flow-mediated dilation (23). Chronic low-grade inflammation, characterized by elevated C-reactive protein and pro-inflammatory cytokines, represents another mechanistic pathway linking hypogonadism to accelerated atherosclerosis (11). Additionally, testosterone deficiency may adversely affect vascular smooth muscle function and arterial compliance, contributing to increased arterial stiffness and elevated blood pressure. The clustering of these metabolic and vascular abnormalities in hypogonadal diabetic men creates a particularly high-risk phenotype for ASCVD development and progression. Although several African studies have documented high hypogonadism prevalence in men with T2DM, only one South African study directly linked low testosterone with prevalent cardiovascular disease, and none have examined the calculated 10-year ASCVD risk in Nigerian men, given potential genetic, environmental, and healthcare system differences (20). Therefore, the study aimed to compare 10-year ASCVD risk and cardiometabolic profiles between T2DM and non-diabetic control men, stratified by testosterone status, and to explore associations between testosterone and cardiovascular risk factors within the T2DM cohort.

## Materials and methods

### Study design and participants

This was a hospital-based cross-sectional study conducted at Ahmadu Bello University Teaching Hospital, Zaria, Nigeria. The study included 537 adult men, comprising 358 with T2DM, recruited consecutively at the endocrine clinic, and 179 non−diabetic controls. Participants were further stratified into low- and normal-testosterone groups, yielding four subgroups: T2DM with normal testosterone, T2DM with low testosterone, non−diabetic controls with normal testosterone, and non−diabetic controls with low testosterone. The inclusion criteria were men with T2DM aged 21 years and above. Exclusion criteria included those under 21, prior or current testosterone or anti-androgen therapy, known hypogonadism, chronic liver disease, chronic kidney disease, panhypopituitarism, HIV infection, acute illness requiring hospitalization, type 1 diabetes, and suspected prostate or testicular malignancy. The study cohort and inclusion criteria have been published previously (9, 18). Ethical approval was obtained from the ABUTH Ethics Committee (ABUTHZ/HREC/NP18/2015), and written informed consent was obtained from all participants.

### Clinical and biological data collection and measurements

Clinical data collected included systolic and diastolic blood pressure (SBP, DBP), weight, height, body mass index (BMI), and waist circumference, all measured using standardized protocols. Sociodemographic and clinical information were obtained using a structured questionnaire and have been published (9, 18). Fasting venous blood samples were collected between 8:00 AM and 10:00 AM. Approximately 10 mL of blood was collected in plain tubes, centrifuged for 10 minutes, and the serum was stored at −20 °C until analysis. Serum total testosterone, fasting plasma glucose, and albumin were measured, while free testosterone was calculated using Vermeulen’s method (24). An additional 5 mL of blood was collected in EDTA tubes for glycated hemoglobin (HbA1c) estimation. Total testosterone was measured using an enzyme-linked immunosorbent assay (Monobind Inc., CA, USA). Low testosterone was defined as total testosterone <12 nmol/L, with values >12 nmol/L considered normal, in accordance with recommendations that total testosterone <8 nmol/L strongly supports a diagnosis of hypogonadism, >12 nmol/L is normal, and 8–12 nmol/L with symptoms suggests mild testosterone deficiency syndrome (25). Although free testosterone was calculated using the Vermeulen equation to complete androgen profiling, these values were not presented as they did not provide additional significant discriminatory or interpretive value for the study beyond total testosterone. Throughout this study, the term testosterone refers specifically to total serum testosterone.

### ASCVD risk estimation

The 10-year ASCVD risk score was calculated for each participant using the ACC/AHA Pooled Cohort Equations based on age, cholesterol levels, blood pressure, diabetes status, and smoking history (26). Because the ACC/AHA Pooled Cohort Equations were developed and validated in U.S. populations, their application to a Nigerian cohort may introduce calibration limitations; therefore, the ASCVD risk estimates in this study should be interpreted with appropriate caution.

### Statistical analysis

Data were analyzed using GraphPad Prism software and Python. Normality was assessed using the Shapiro–Wilk test. Continuous variables were expressed as mean ± standard deviation. Two-way ANOVA was used to analyze the cardiometabolic profile of men according to T2DM status and testosterone levels. Pearson correlation analysis was used to assess the associations between the ASCVD risk score and cardiovascular risk factors, and testosterone levels and HbA1c. Multivariable regression and correlation analyses were performed within the T2DM cohort, adjusting for confounding factors, including age, BMI, waist circumference and HbA1c. Cohen’s d for all pairwise subgroup comparisons and partial η² for the two−factor (T2DM × testosterone) ANOVA were computed. All effect−size estimates were calculated on the same models that adjust for age, BMI, HbA1c, and waist circumference. A p-value <0.05 was considered statistically significant, and P_T2DM_ represents the overall statistically significant effect of T2DM on the parameters.

## Results

**Figure 1:**
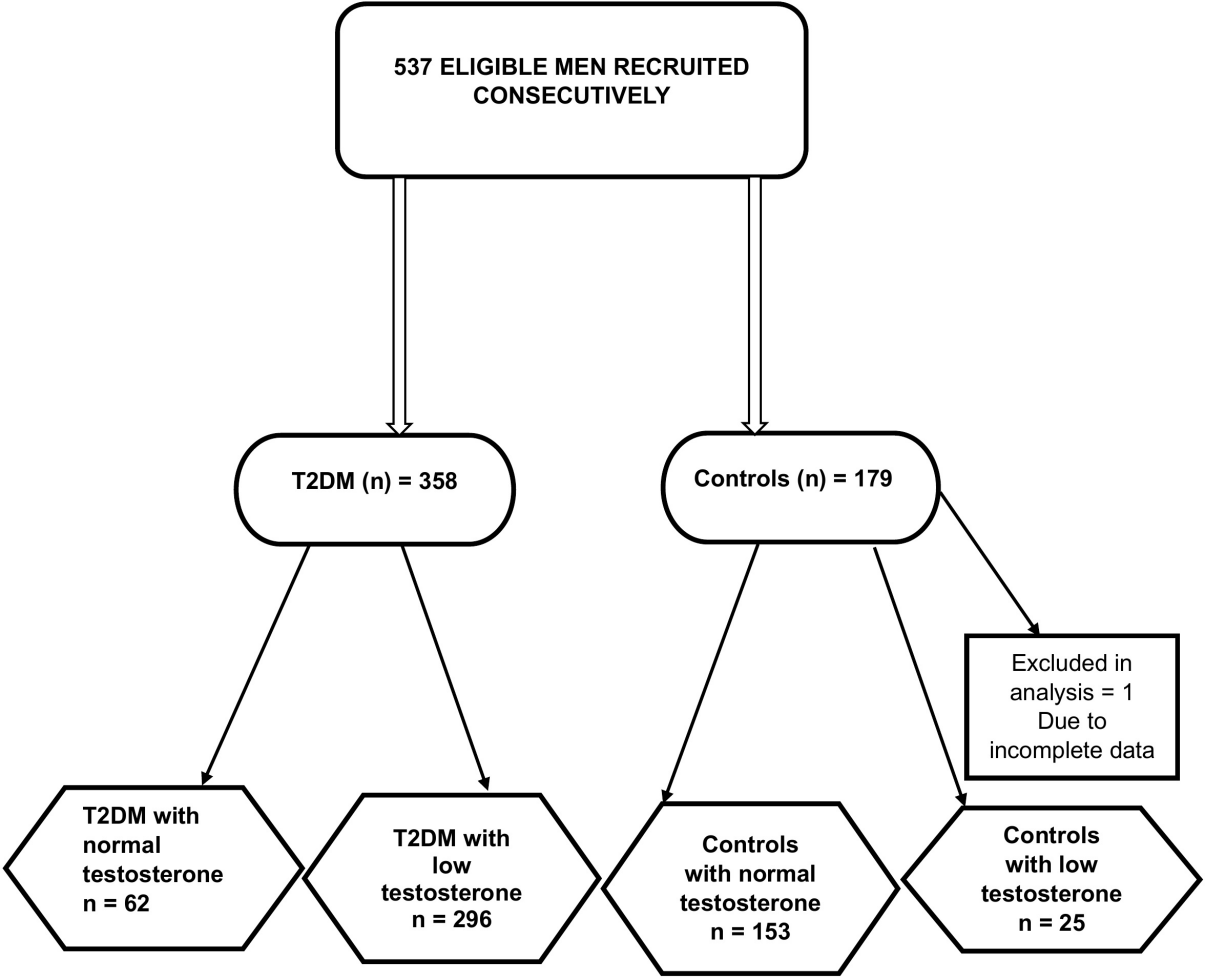
Flow chart of the study participants.

A total of 358 men with T2DM and 178 healthy men as non−diabetic controls were included in the final analysis, stratified further by testosterone status into normal and low testosterone subgroups. Although 179 non−diabetic men were initially enrolled, data from only 178 were analyzed due to missing data for one individual. Within the T2DM group, 62 had normal testosterone, and 296 had low testosterone; in the non−diabetic controls group, 153 had normal testosterone, and 25 had low testosterone (**Figure 1**). Participants with T2DM were notably older than non−diabetic controls across both testosterone subgroups, with mean ages of 56.32 ± 9.90 and 57.08 ± 10.75 years in the T2DM normal- and low-testosterone groups, respectively, compared with 39.02 ± 13.94 and 40.84 ± 11.85 years in the corresponding non−diabetic control groups (P_T2DM_<0.0001, P_Testosterone_<0.0001). For effect sizes reporting, the partial η² for the main effect of low testosterone on the 10−year ASCVD score was 0.004 (trivial; p = 0.14), whereas the partial η² for T2DM status on ASCVD was 0.221 (large). Cohen’s d for ASCVD between the T2DM−Low and T2DM−Normal testosterone subgroups was 0.08 (trivial), while Cohen’s d for comparisons between T2DM and non−diabetic men were large for ASCVD (d = 1.67), HbA1c (d = 2.27) and waist circumference (d = 1.14) as seen in **Supplementary Tables 1A–C**.

### Cardiometabolic profile of men according to T2DM status and testosterone levels

**Figure 2:**
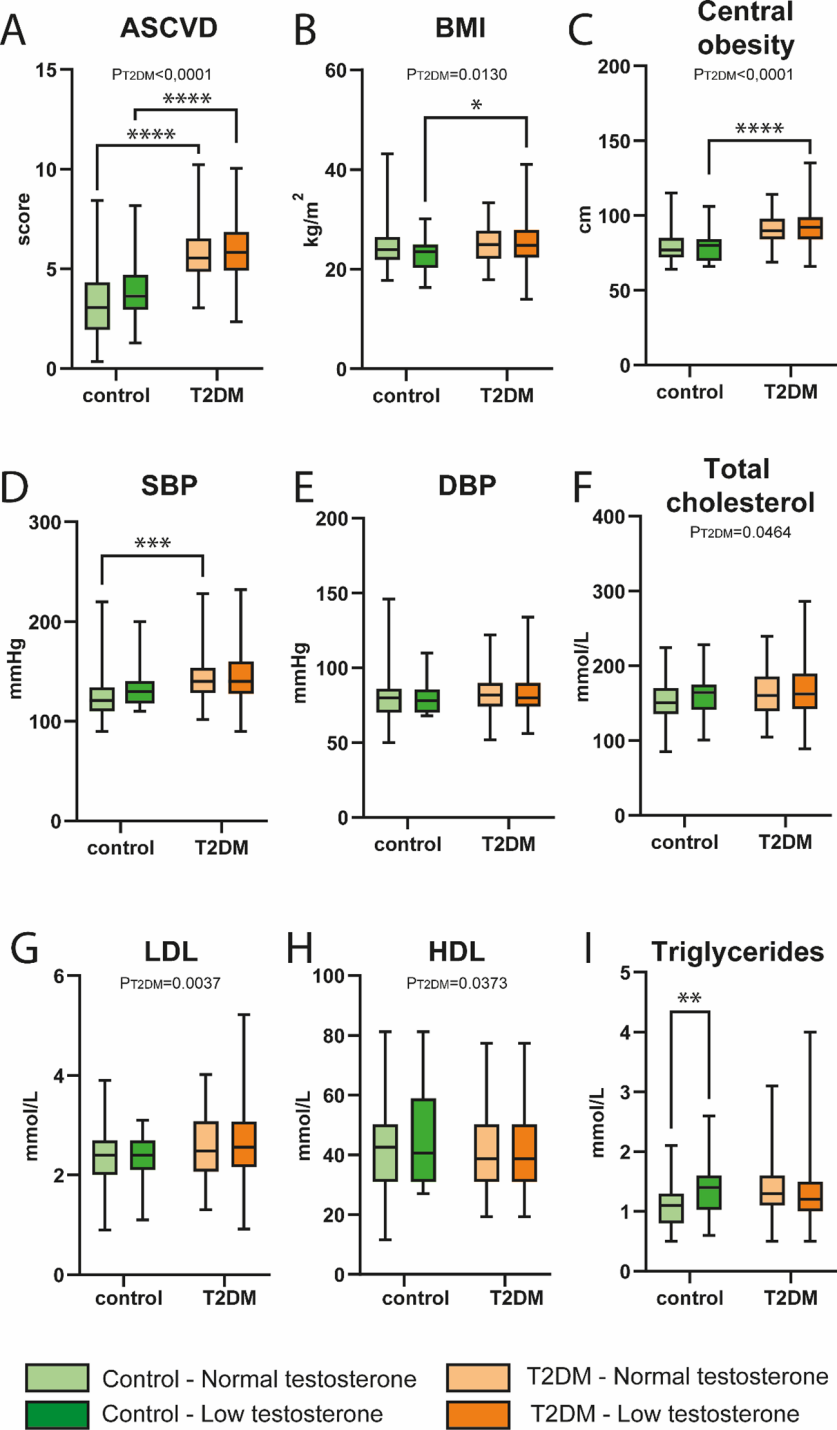
Cardiometabolic profile of men according to T2DM status and testosterone levels. Box-and-whisker plots depicting **(A)** 10-year atherosclerotic cardiovascular disease (ASCVD) risk score, **(B)** body mass index (BMI), **(C)** central obesity (waist circumference), **(D)** systolic blood pressure (SBP), **(E)** diastolic blood pressure (DBP), **(F)** total cholesterol, **(G)** low-density lipoprotein (LDL) cholesterol, **(H)** high-density lipoprotein (HDL) cholesterol, and **(I)** triglycerides in men without diabetes (control) and with type 2 diabetes mellitus (T2DM), stratified by testosterone status. Non−diabetic controls with normal testosterone (light green), non−diabetic controls with low testosterone (dark green), T2DM men with normal testosterone (light orange), and T2DM men with low testosterone (dark orange). P_T2DM_ values refer to the overall statistically significant effect of T2DM on the parameters, with asterisks indicating the level of statistical significance between groups (*P<0.05, **P<0.01, ***P<0.001, ****P<0.0001).

This study examined the cardiometabolic profile of men according to T2DM status and testosterone levels. The study found that men with T2DM exhibited significantly higher ASCVD risk than non−diabetic men, regardless of testosterone levels (P_T2DM_<0.0001). Both T2DM men with normal and low testosterone levels had significantly higher ASCVD risk scores than their respective non−diabetic men (**Figure 2A**). BMI differed significantly between the groups (P_T2DM_=0.0130), with eugonadal and hypogonadal men with T2DM having higher BMI values than non−diabetic controls. Notably, the T2DM group with low testosterone tended to have significantly higher BMI compared to non−diabetic controls with low testosterone. Central obesity, assessed by waist circumference, was significantly higher in T2DM men than in their non−diabetic counterparts, irrespective of testosterone status (P_T2DM_ < 0.0001). T2DM men with low testosterone exhibited markedly greater central obesity (**Figures 2B, C**) compared to the non−diabetic controls with low testosterone. SBP was significantly higher in individuals with T2DM men (P_T2DM_=0.0002) with normal testosterone concentrations than in non−diabetic controls with normal testosterone (P<0.001). Mean SBP was higher in both T2DM subgroups, trending toward hypertensive ranges, particularly among men with low testosterone. DBP did not differ significantly between the T2DM groups and the non−diabetic controls (**Figures 2D, E**). Total cholesterol levels were significantly elevated in T2DM men (P_T2DM_=0.0464) compared with non−diabetic controls. LDL cholesterol followed a similar pattern, with higher levels in men with T2DM (P_T2DM_=0.0037) than in the non−diabetic controls. Conversely, HDL cholesterol showed an inverse trend, showing a significant overall difference (P_T2DM_=0.0373), with T2DM groups demonstrating lower HDL cholesterol than non−diabetic controls. Serum triglyceride levels were significantly higher in control men with low testosterone compared to their non−diabetic counterparts with normal testosterone (P<0.01) (**Figures 2F–I**).

### Correlation between cardiometabolic risk factors with testosterone levels and HbA1c in men with and without T2DM

**Figure 3:**
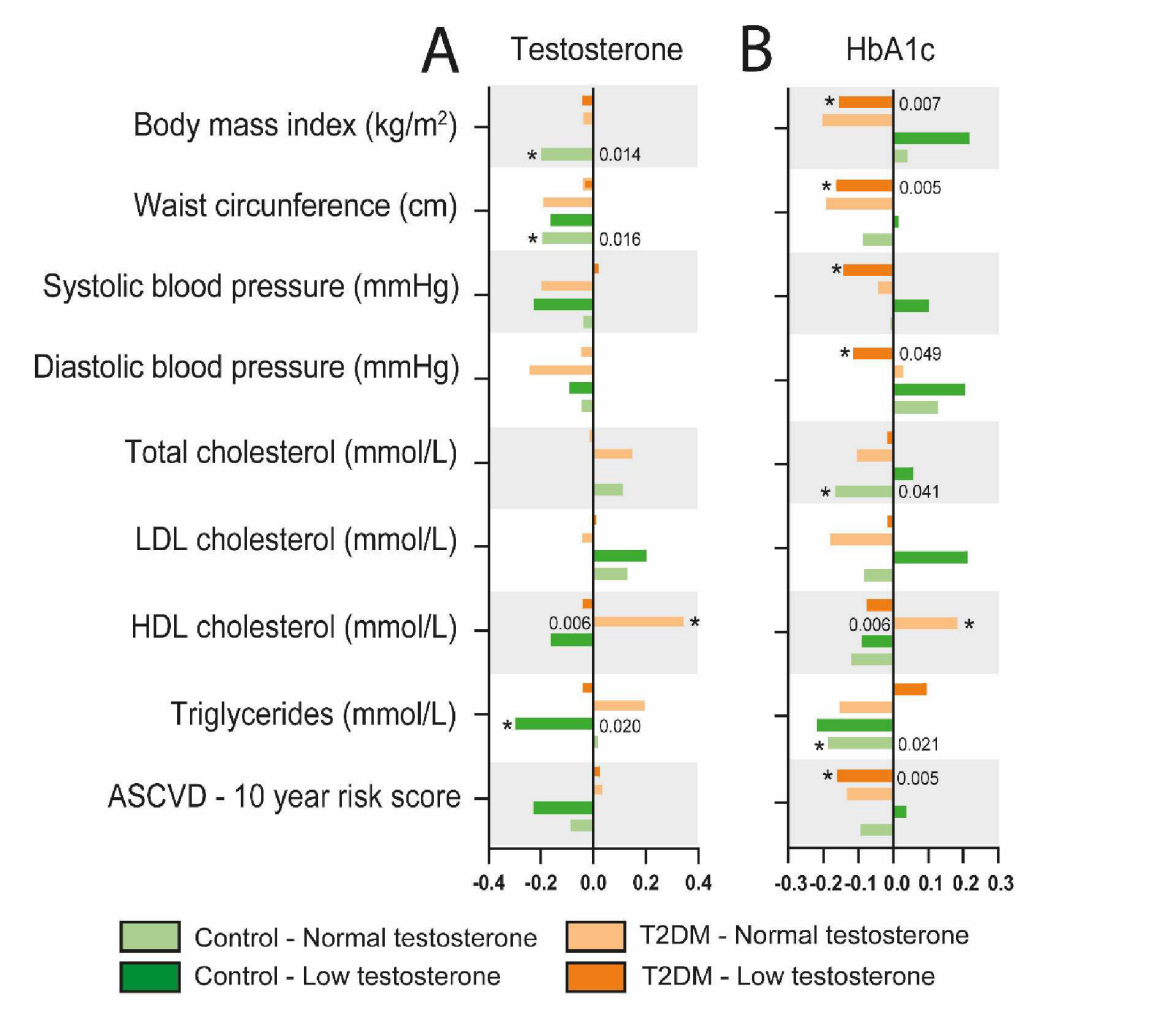
Correlations between cardiometabolic risk factors and testosterone and HbA1c in men with and without T2DM. Correlations of serum testosterone **(A)** and HbA1c **(B)** with body mass index (BMI), waist circumference, systolic and diastolic blood pressure, total cholesterol, low−density lipoprotein (LDL) cholesterol, high−density lipoprotein (HDL) cholesterol, triglycerides, and 10−year atherosclerotic cardiovascular disease (ASCVD) risk score. Non−diabetic controls with normal testosterone (light green), non−diabetic controls with low testosterone (dark green), T2DM men with normal testosterone (light orange), and T2DM men with low testosterone (dark orange). Bars indicate the direction and magnitude of the standardized correlation **(r)** for each cardiometabolic risk factor, adjusted for relevant covariates. Asterisks denote statistically significant associations (typically *P<0.05), with illustrative P−values given alongside selected bars.

BMI and waist circumference showed significant inverse relationships with testosterone levels in non−diabetic eugonadal men (r: -0.199, P = 0.014; r: -0.194, P = 0.016, respectively) but paradoxically showed negative correlations with HbA1c in T2DM men with low testosterone (r: -0.155, P = 0.007; r: -0.164, P = 0.005, respectively). For blood pressure, both SBP and DBP were negatively associated with HbA1c in T2DM men with low testosterone (r: -0.145, P = 0.012; r: -0.114, P = 0.049, respectively). Total cholesterol, which showed a significant inverse relationship with HbA1c among eugonadal non−diabetic controls (r: -0.166, P = 0.041). There was a significant positive relationship between HDL cholesterol and both testosterone levels and HbA1c in T2DM men with normal testosterone (r: 0.348, P = 0.006; r: 0.183, P = 0.006, respectively). Triglycerides showed negative associations with testosterone and HbA1c in non−diabetic men with low and normal testosterone, respectively (r: -0.299, P = 0.020; r: -0.187, P = 0.021, respectively). HbA1c was negatively associated with ASCVD 10-year risk in T2DM men with low testosterone (r: -0.133, P = 0.005) (**Figure 3**).

### Multivariable regression and correlation analyses between serum testosterone and cardiometabolic risk factors in T2DM men

In multivariable models within the T2DM cohort, adjusted for age, BMI, waist circumference and HbA1c, serum testosterone showed no independent association with the ASCVD risk score, blood pressure, or cholesterol fractions (all P > 0.05; **Tables 1**, **2**).

**Table 1:** Multivariable regression analysis between serum testosterone and cardiometabolic risk factors in T2DM men.

Outcome	T2DM men (n = 358) Multivariable regression coefficient (β) (95% CI)	P value
ASCVD score	−0.004 (−0.031, 0.023)	0.78
SBP	+0.052 (−0.714, 0.817)	0.89
DBP	−0.240 (−0.644, 0.164)	0.24
Total cholesterol	−0.006 (−0.034, 0.023)	0.70
LDL cholesterol	−0.005 (−0.028, 0.018)	0.67
HDL cholesterol	−0.037 (−0.434, 0.360)	0.85
Triglycerides	+0.001 (−0.014, 0.016)	0.92

ASCVD, atherosclerotic cardiovascular disease, DBP, diastolic blood pressure, HDL, high density lipoprotein, LDL, low density lipoprotein, SBP, systolic blood pressure. All models adjusted for age, BMI (body mass index), waist circumference and HbA1c (glycated hemoglobin).

**Table 2:** Multivariable correlation analysis between serum testosterone and cardiometabolic risk factors in T2DM men.

Outcome	T2DM men (n = 358) Multivariable correlation (r)	P value
ASCVD score	−0.015	0.78
SBP	+0.006	0.91
DBP	−0.061	0.25
Total cholesterol	−0.019	0.72
LDL cholesterol	−0.021	0.69
HDL cholesterol	−0.010	0.85
Triglycerides	+0.007	0.90

ASCVD, atherosclerotic cardiovascular disease, DBP, diastolic blood pressure, HDL, high density lipoprotein, LDL, low density lipoprotein, SBP, systolic blood pressure. All models adjusted for age, BMI (body mass index), waist circumference and HbA1c (glycated hemoglobin).

## Discussion

Nigerian men with T2DM, particularly those with concomitant low testosterone, showed the highest 10-year ASCVD risk and the most adverse cardiometabolic profile, although this association did not persist after multivariate adjustment. The study demonstrates that T2DM men with low testosterone showed significant cardiometabolic derangements, with deterioration evident across all measured parameters, including anthropometric, hemodynamic, and lipid profiles, compared with eugonadal men with T2DM. This study adds to the limited evidence on testosterone status and cardiovascular risk among African men with T2DM, which has been historically underrepresented in testosterone and cardiovascular studies. The findings highlight a complex relationship between androgen deficiency and cardiometabolic risk, providing insights that could inform clinical practice and strategies for managing T2DM.

The clustering of the highest ASCVD scores in hypogonadal T2DM men is consistent with data from a prospective cohort, which demonstrated that declining testosterone was associated with significantly higher estimated 10-year cardiovascular risk, and with a meta-analysis showing a trend toward higher coronary artery disease risk in T2DM men with low testosterone (27). Furthermore, a large meta-analysis established that lower testosterone is independently associated with increased cardiovascular mortality (28). In the Nigerian context, studies reported hypogonadism prevalence rates of 80.4% and 52.5%, respectively, among men with T2DM, underscoring the magnitude of this compounded risk in this population (9, 29). In contrast, a meta-analysis did not reach statistical significance in its primary analysis, and certain studies in chronic stable coronary artery disease have paradoxically reported higher testosterone levels associated with greater coronary stenosis, suggesting that the testosterone-ASCVD relationship is likely non-linear and context-dependent (14). The significant negative associations between adiposity indices and HbA1c among hypogonadal men with T2DM reveal a distinct diabetes-driven pathophysiological subtype (30, 31). Mechanistically, visceral adipose tissue expansion upregulates aromatase, converting testosterone to estradiol and suppressing the hypothalamic–pituitary–gonadal (HPG) axis; adipose-derived leptin and tumor necrosis factor-α further inhibit gonadotropin-releasing hormone secretion, perpetuating a self-reinforcing hypogonadal–obesity cycle. Waist circumference has been shown to be a stronger independent determinant of hypogonadism than HbA1c in middle-aged men, yet in hypogonadal T2DM men, chronic hyperglycemia appears to independently sustain adiposity through Leydig cell glucotoxicity and HPG dysregulation (32). This finding implies that optimizing glycemic control may confer a secondary benefit of partially restoring androgen levels, beyond its direct effect on adiposity. The consistently higher systolic blood pressure (SBP) in T2DM subgroups, particularly in hypogonadal men, corroborates evidence that lower serum testosterone is associated with impaired flow-mediated dilation, a marker of endothelial dysfunction independent of classical cardiovascular risk factors (33, 34). The underlying mechanism involves androgen receptor-dependent activation of endothelial nitric oxide synthase (eNOS), the loss of which reduces nitric oxide (NO) bioavailability, promoting vasoconstriction and systemic hypertension (35). The inverse association between SBP and DBP and HbA1c in hypogonadal T2DM men may reflect a therapeutic confound: patients with higher HbA1c may receive more intensive antihypertensive or glucose-lowering therapy, including agents with vasodilatory properties, thereby attenuating blood pressure. This observation warrants prospective evaluation after accounting for medication use.

The atherogenic lipid profile characterizing T2DM men in this cohort, elevated total and LDL cholesterol, and reduced HDL, is consistent with the established metabolic dyslipidemia of diabetes. The positive association between HDL cholesterol and testosterone observed in T2DM men with normal testosterone is consistent with the known dose-dependent effects of testosterone on HDL: at physiological levels, testosterone modulates HDL protein composition, increasing the abundance of paraoxonase-1 (PON1), an antioxidant HDL-associated enzyme with anti-atherogenic properties (36). The paradoxical HDL–HbA1c co-elevation in hypogonadal T2DM men may reflect differential use of medications that raise HDL cholesterol among patients receiving intensified therapy, and does not imply any protective effect of hyperglycemia (36) (37),. Future studies should consider medication-adjusted analyses to further investigate this relationship. The significant negative association between HbA1c and 10-year ASCVD risk in hypogonadal T2DM men is clinically counterintuitive but not unprecedented. The ASCVD Pooled Cohort Equation does not incorporate HbA1c; it weighs age, sex, systolic blood pressure, lipid profile, antihypertensive treatment, and smoking. Consequently, men with higher HbA1c but a favorable constellation of the remaining inputs, notably younger age, lower treated BP, and non-smoking status, can simultaneously exhibit poor glycemic control and lower calculated ASCVD risk. HbA1c-associated CVD risk is significantly modified by baseline ASCVD stratum, with elevated HbA1c conferring substantially higher CVD risk only in patients with moderate, not low, baseline ASCVD risk (HR 2.48; 95% CI 1.15–5.32) (38). This interaction may explain the seemingly protective relationship observed; rather than implying a cardioprotective role for hyperglycemia, it reflects the dominance of non-glycemic ASCVD risk drivers in this specific subgroup and reinforces the need for integrated, multi-factorial cardiovascular risk assessment beyond HbA1c alone.

While unadjusted differences might suggest a role for testosterone in cardiovascular risk assessment, our adjusted analyses using multivariable regression and correlation analyses within the T2DM cohort indicate that testosterone was not independently associated with the ASCVD risk score, adiposity indices, blood pressure, or lipid fractions after adjustment for age, BMI, waist circumference, and HbA1c. The evidence that testosterone is an independent predictor of ASCVD or cardiometabolic risk in men with established T2DM is inconsistent. Studies have suggested that low testosterone may contribute to atherosclerotic vascular disease and adverse cardiometabolic risk in men with T2DM. Low testosterone in men with T2DM was associated with more advanced subclinical atherosclerotic disease, including increased carotid intima-media thickness and endothelial dysfunction, even after adjusting for age, HbA1c, lipids, treatment effect, and BMI (23). Furthermore, low testosterone level was associated with cardiovascular events in Japanese men, independent of coronary risk factors and endothelial function, while another study reported low testosterone as an independent predictor of the severity of coronary artery disease (39, 40). However, similar to our findings, a meta-analysis in men with T2DM found that lower testosterone was not significantly associated with a higher risk of coronary artery disease/cardiovascular disease, and prospective population data from the FINRISK97 study found that low testosterone did not predict incident coronary heart disease (14, 41). Testosterone may exert direct cardiovascular effects, such as modulation of endothelial function, vascular tone, and myocardial electrophysiology, as described in prior mechanistic studies (42). However, it may also influence cardiovascular risk indirectly through metabolic pathways involving adiposity, insulin sensitivity, and lipid handling (43, 44). Our data cannot distinguish between these pathways, and the associations reported here should therefore be interpreted strictly as correlations. Furthermore, the interaction between testosterone and cardiometabolic risk may differ in African populations due to race−specific physiological characteristics. African men have been shown to exhibit distinct androgen profiles, lower visceral adiposity at comparable BMI, and differing lipid distributions relative to other ethnic groups (45, 46), all of which may modify the relationship between testosterone and cardiovascular risk. The limited availability of longitudinal testosterone–cardiovascular data in African cohorts underscores the need for population−specific studies to clarify these complex interactions.

This study has limitations. First, the cross-sectional design precludes causal inference; reverse causality, whereby existing cardiovascular risk factors suppress testosterone, cannot be excluded. Second, the ASCVD Pooled Cohort Equations were derived in US-based cohorts; their applicability to continental West African populations requires validation, as comorbidity burden, risk factor prevalence, and cardiovascular event rates differ substantially from those in African American reference populations (14, 47). Third, a single-center, tertiary hospital, consecutive recruitment strategy may introduce selection bias and limit generalizability to the broader Nigerian diabetic population. Fourth, in our study, there was an age imbalance between groups, which is relevant to both testosterone physiology and cardiovascular risk estimation and could impact the interpretation of some results. However, we have adjusted for age in all multivariable regression and correlational analyses, ensuring that the associations reported in the manuscript account for the potential confounding effect of age. Given that the ACC/AHA Pooled Cohort Equations (PCE) were developed and validated in U.S. cohorts, this may affect risk estimation in our Nigerian sample, as several studies conducted in sub−Saharan Africa have demonstrated systematic overestimation of absolute 10−year ASCVD risk when the PCE are applied to African populations. For example, a substantial overprediction of ASCVD risk was reported among Ghanaian migrants and non−migrants (48). Similarly, U.S.−derived equations overestimated cardiovascular risk in South African cohorts and performed less accurately than locally derived models (49). More recently, a study showed that the PCE overestimated risk in a Ugandan population and recommended cautious interpretation of absolute scores (50). To address this limitation, we suggest that, in the present study, the ASCVD score be used primarily as a risk−stratification tool rather than as a precise predictor of 10−year event probability.

This study provides the first testosterone-stratified evaluation of estimated 10-year ASCVD risk in Nigerian men with T2DM. T2DM was associated with substantially higher estimated 10-year ASCVD risk than non-diabetic controls. Among men with T2DM, low testosterone was associated with a more adverse cardiometabolic profile. However, after multivariable adjustment, serum testosterone was not independently associated with estimated ASCVD risk or individual cardiometabolic risk factors. These findings suggest that testosterone status may serve as a marker of cardiometabolic phenotype rather than an independent ASCVD risk predictor in men with established T2DM. Prospective studies in African populations are needed to determine whether testosterone improves cardiovascular risk prediction or identifies patients who may benefit from targeted intervention.

## Data Availability

The raw data supporting the conclusions of this article will be made available by the authors, without undue reservation.
