# Supplementary material for: Serum testosterone and cardiometabolic risk in Nigerian men with type 2 diabetes: a cross−sectional study
Source: Front Endocrinol (Lausanne). 2026 Jun 16;17:1843249. doi: 10.3389/fendo.2026.1843249 (PMC13314498; doi:10.3389/fendo.2026.1843249)
Supplement: Supplementary file 1 [file Table1.docx]

*Supplementary material — Manuscript 1843249*

**Serum Testosterone and Cardiometabolic Risk in Nigerian Men with Type 2 Diabetes: A Cross‑Sectional Study**

# Supplementary Table S1 — Effect sizes

Effect sizes were computed to complement the p-values from the two-factor (T2DM × testosterone) ANOVA and to address the subgroup-size imbalance flagged by Reviewer 2 (T2DM-normal n = 62 vs T2DM-low n = 296; control-normal n = 153 vs control-low n = 25). Partial η² is reported for each main effect and the interaction in the 2 × 2 ANOVA; Cohen’s d is reported for pairwise contrasts between the four subgroups. Conventional thresholds are used for interpretation: |d| < 0.2 trivial, 0.2–0.5 small, 0.5–0.8 medium, > 0.8 large; partial η² 0.01 small, 0.06 medium, 0.14 large.

## Panel A — Baseline characteristics by subgroup (mean ± SD)

| **Variable** | **Control-Normal (n=152)** | **Control-Low (n=26)** | **T2DM-Normal (n=60)** | **T2DM-Low (n=298)** |
| --- | --- | --- | --- | --- |
| **Age (y)** | 38.8 ± 13.8 | 41.9 ± 12.7 | 56.6 ± 9.9 | 57.0 ± 10.7 |
| **SBP (mmHg)** | 127.6 ± 22.5 | 135.8 ± 22.7 | 143.5 ± 23.2 | 143.3 ± 26.1 |
| **DBP (mmHg)** | 79.7 ± 14.4 | 80.9 ± 11.9 | 81.9 ± 11.7 | 82.7 ± 13.3 |
| **BMI (kg/m²)** | 24.5 ± 3.8 | 23.1 ± 3.5 | 25.0 ± 3.8 | 25.3 ± 4.6 |
| **Waist (cm)** | 79.5 ± 9.8 | 79.1 ± 10.6 | 90.8 ± 9.6 | 92.7 ± 12.4 |
| **HbA1c (%)** | 5.09 ± 0.71 | 4.93 ± 0.91 | 9.21 ± 2.15 | 9.11 ± 2.11 |
| **Total chol (mmol/L)** | 3.95 ± 0.68 | 4.12 ± 0.82 | 4.21 ± 0.85 | 4.30 ± 0.94 |
| **LDL (mmol/L)** | 2.37 ± 0.57 | 2.32 ± 0.49 | 2.55 ± 0.65 | 2.65 ± 0.76 |
| **HDL (mmol/L)** | 1.09 ± 0.32 | 1.18 ± 0.43 | 1.04 ± 0.33 | 1.05 ± 0.33 |
| **Triglycerides (mmol/L)** | 1.07 ± 0.32 | 1.37 ± 0.51 | 1.35 ± 0.48 | 1.32 ± 0.49 |
| **Testosterone (nmol/L)** | 16.6 ± 2.2 | 8.1 ± 2.9 | 13.7 ± 1.4 | 7.8 ± 2.7 |
| **ASCVD 10-year score** | 3.31 ± 1.66 | 3.90 ± 1.53 | 5.74 ± 1.35 | 5.86 ± 1.46 |

*Age is reported in years. Lipid units as specified. Testosterone in nmol/L (low testosterone threshold < 12 nmol/L).*

## Panel B — Cohen’s d for pairwise contrasts

| **Outcome** | **T2DM-Low vs T2DM-Norm** | **T2DM-Low vs Control-Norm** | **T2DM-Low vs Control-Low** | **Control-Low vs Control-Norm** |
| --- | --- | --- | --- | --- |
| **ASCVD score** | 0.08 | 1.67 | 1.34 | 0.36 |
| **BMI** | 0.08 | 0.19 | 0.50 | −0.38 |
| **Waist (cm)** | 0.17 | 1.14 | 1.11 | −0.04 |
| **SBP** | −0.01 | 0.63 | 0.29 | 0.37 |
| **DBP** | 0.06 | 0.22 | 0.14 | 0.08 |
| **HbA1c** | −0.05 | 2.27 | 2.04 | −0.21 |
| **Total cholesterol** | 0.10 | 0.41 | 0.19 | 0.25 |
| **HDL** | 0.01 | −0.13 | −0.39 | 0.27 |
| **LDL** | 0.14 | 0.40 | 0.45 | −0.09 |
| **Triglycerides** | −0.07 | 0.57 | −0.11 | 0.86 |
| **Testosterone** | −2.31 | −3.46 | −0.12 | −3.66 |

*Positive values indicate a higher mean in the first-named group. For testosterone, negative values reflect the expected lower levels in the “Low” subgroups. Cohen’s d between T2DM-Low and T2DM-Normal men is trivial (|d| ≤ 0.17) for all cardiometabolic outcomes, indicating that within T2DM men the hypogonadal vs eugonadal contrast carries little additional clinical information once T2DM status is known.*

## Panel C — Partial η² from two-way ANOVA

| **Outcome** | **T2DM main effect η²p** | **p** | **Low-T main effect η²p** | **p** | **Interaction η²p** |
| --- | --- | --- | --- | --- | --- |
| **ASCVD score** | 0.221 | <0.0001 | 0.004 | 0.14 | 0.003 |
| **BMI** | 0.008 | 0.036 | 0.000 | 0.71 | 0.005 |
| **Waist** | 0.125 | <0.0001 | 0.002 | 0.35 | 0.001 |
| **HbA1c** | 0.404 | <0.0001 | 0.001 | 0.57 | 0.000 |
| **SBP** | 0.034 | <0.0001 | 0.001 | 0.41 | 0.003 |
| **Total chol.** | 0.009 | 0.029 | 0.003 | 0.25 | 0.000 |
| **HDL** | 0.007 | 0.061 | 0.001 | 0.44 | 0.002 |
| **LDL** | 0.014 | 0.006 | 0.001 | 0.48 | 0.001 |
| **Triglycerides** | 0.017 | 0.003 | 0.003 | 0.19 | 0.016 |
| **Testosterone** | 0.077 | <0.0001 | 0.498 | <0.0001 | 0.032 |

*The partial η² values for the main effect of low-testosterone on ASCVD score (0.004), BMI (0.000), waist (0.002), HbA1c (0.001), and blood pressure are trivial and non-significant. In contrast, T2DM status shows large effects on HbA1c (0.40), ASCVD (0.22), and waist (0.13). A small-to-moderate significant interaction is present for triglycerides (η²p = 0.016, p = 0.003), indicating the testosterone-triglyceride relationship differs between diabetic and non-diabetic men.*

# Key methodological points

• Analyses were performed in Python (pandas, statsmodels, scipy) on the de-identified study dataset (n = 536 after exclusion of one control with incomplete data). Scripts are available from the corresponding author on reasonable request.

• Two-way ANOVA was fit with Type II sum of squares (factors: T2DM status, testosterone status, and their interaction). Partial η² was computed as SS_effect / (SS_effect + SS_residual).

• Cohen’s d used the pooled standard deviation of the two groups being compared.

• Multivariable linear regression (main text) used ordinary least squares. Model residuals were examined; no severe violations of linearity or homoscedasticity were detected that would alter the substantive conclusions.
